# Characterisation of clot microstructure properties in stable coronary artery disease

**DOI:** 10.1136/openhrt-2016-000562

**Published:** 2017-06-23

**Authors:** Ahmed Sabra, Matthew James Lawrence, Robert Aubrey, Daniel Obaid, Alexander Chase, Dave Smith, Phillip Thomas, Sharon Storton, Gareth R Davies, Karl Hawkins, Phylip Rhodri Williams, Keith Morris, Phillip Adrian Evans

**Affiliations:** 1 NISCHR Haemostasis Biomedical Research Unit, Morriston Hospital, ABMU Health Board, Swansea, UK; 2 NISCHR Haemostasis Biomedical Research Unit, College of Medicine, Swansea University, Swansea, UK; 3 Department of Cardiology, Princess of Wales Hospital, ABMU Health Board, Bridgend, UK; 4 Cardiac Centre, Morriston Hospital, ABMU Health Board, Swansea, UK; 5 College of Engineering, Swansea University, Swansea, UK; 6 School of Applied Science, Cardiff Metropolitan University, Cardiff, UK; 7 Department of Emergency Medicine, Morriston Hospital, ABMU Health Board, Swansea, UK

**Keywords:** atherosclerosis, coronary artery disease, coronary angiography, coagulation, clot microstructure

## Abstract

**Background:**

Coronary artery disease (CAD) is associated with an increased prothrombotic tendency and is also linked to unfavourably altered clot microstructure. We have previously described a biomarker of clot microstructure (d_f_) that is unfavourably altered in acute myocardial infarction. The d_f_ biomarker assesses whether the blood will form denser or looser microstructures when it clots. In this study we assessed in patients with stable chest pain whether d_f_ can differentiate between obstructed and unobstructed CAD.

**Methods:**

A blood sample prior to angiography was obtained from 251 consecutive patients undergoing diagnostic coronary angiography. Patients were categorised based on angiographic findings as presence or absence of obstructive CAD (stenosis ≥50%). The blood sample was assessed using the d_f_ biomarker, standard laboratory markers and platelet aggregometry (Multiplate).

**Results:**

A significant difference (p=0.028) in d_f_ was observed between obstructive CAD (1.748±0.057, n=83) and unobstructive CAD (1.732±0.052, n=168), where patients with significant CAD produce denser, more tightly packed clots. d_f_ was also raised in men with obstructive CAD compared with women (1.745±0.055 vs 1.723±0.052, p=0.007). Additionally d_f_ significantly correlated with the platelets response to arachidonic acid as measured by the ASPItest area under the curve readings from platelet aggregometry (correlation coefficient=0.166, p=0.008), a low value of the ASPItest indicating effective aspirin use was associated with looser, less dense clots.

**Conclusions:**

For the first time, we characterise clot microstructure, as measured by d_f_, in patients with stable CAD. d_f_ can potentially be used to risk-stratify patients with stable CAD and assess the efficacy of therapeutic interventions by measuring changes in clot microstructure.

**Key questions**

**What is already known about this subject?**

Coronary artery disease (CAD) alters coagulation and is associated with an increased risk of thrombotic disease.

**What does this study add?**

We show how a novel marker of clot microstructure can be used to characterise the level of disease in stable CAD and therapeutic manipulation.

**How might this impact on clinical practice?**

We identify a possible tool for risk-stratifying patients with stable CAD, alongside the potential to assess the efficacy of therapeutic interventions.

## Introduction

Coronary artery disease (CAD) is associated with an underlying systemic imbalance in haemostasis caused by the presence of a hypercoagulable state and a decrease in fibrinolysis.^1–4^ While CAD has been linked to an increased prothrombotic state, no marker has been identified that can accurately assess abnormalities of global haemostasis due to this process and to severity of disease. Identifying a global haemostatic marker of coagulability and fibrinolysis may be important in stratifying risk of atherothrombosis and providing the basis for individualised therapeutic management.

Previous studies have identified that abnormal clot microstructure is of significant importance in the pathophysiology of many vascular and inflammatory disease states including CAD.^5–8^ However, the standard techniques for assessing clot microstructure do not translate to being used as routine markers in a clinical setting.^9^ This has led to the development of a technique that uses assessment of the viscoelastic properties of coagulating blood to quantify its clot microstructure as a fractal dimension, d_f_.^9^ In contrast to standard coagulation assays, the d_f_ measurement is performed using unadulterated whole blood in a near patient setting and provides rapid assessment of coagulation.^9^ Lower values of d_f_ correspond to less dense, less branched, weaker clots, whereas higher d_f_ values represent denser, more complex, stronger clots.^9^ The d_f_ measurement has been previously validated in several disease states and has also been used to stratify the severity of disease, however, its role in stable CAD remains unclear.^10–14^

The aim of the present study was to characterise clot microstructure in CAD. The hypothesis was that for a cohort of patients with suspected CAD undergoing diagnostic angiography, d_f_ will be unfavourably altered in those patients with obstructive CAD compared with those with no or unobstructive CAD.

## Methods

### Patient population

This study was conducted in accordance with good clinical practice and has been reviewed and approved by the by the local Research Ethics Committee (Wales REC 7). We screened all consecutive patients undergoing routine diagnostic coronary angiography for evaluation of new onset chest pain, who have no previously confirmed CAD. Eligible patients were recruited from two hospitals in South Wales (a large teaching hospital and a district general hospital) from November 2012 to August 2014. We excluded patients with active cancer; liver disease; chronic kidney disease stage IV and V or on dialysis; <18 years of age; known clotting disorders; history of myocardial infarction, stroke or thromboembolic disease; severe heart failure (ejection fraction <35% or clinically New York Heart Association (NYHA) stage III–IV) or taking anticoagulants at the time of the study. Written informed consent was obtained from all patients before recruitment in the study.

One venous blood sample was collected before angiography. Data including demographics, medical history and current medications were collected for each patient, including presence of diabetes, family history of CAD (history of acute myocardial in a first-degree relative), hypercholesterolaemia (total cholesterol >5 mmol/L or currently on medication for high cholesterol) and smoking history.

Patients were divided in two groups based on coronary angiographic findings: those with angiographically normal arteries or minor irregularities but no significant stenosis (≥50%) were termed unobstructed, those with any coronary stenosis ≥50% being defined as obstructive CAD. Clinicians reporting coronary angiography findings were blinded to the results of biomarker analysis and the operator performing biomarker analysis was blinded to the angiography results.

### Blood sampling

Each blood sample was divided into several aliquots. One aliquot of whole venous blood was immediately transferred and used for viscoelastic measurements. The remaining aliquots were used to perform standard coagulation screens, full blood count, thrombin generation or platelet aggregometry (see below).

### Viscoelastic measurements

**Figure 1:**
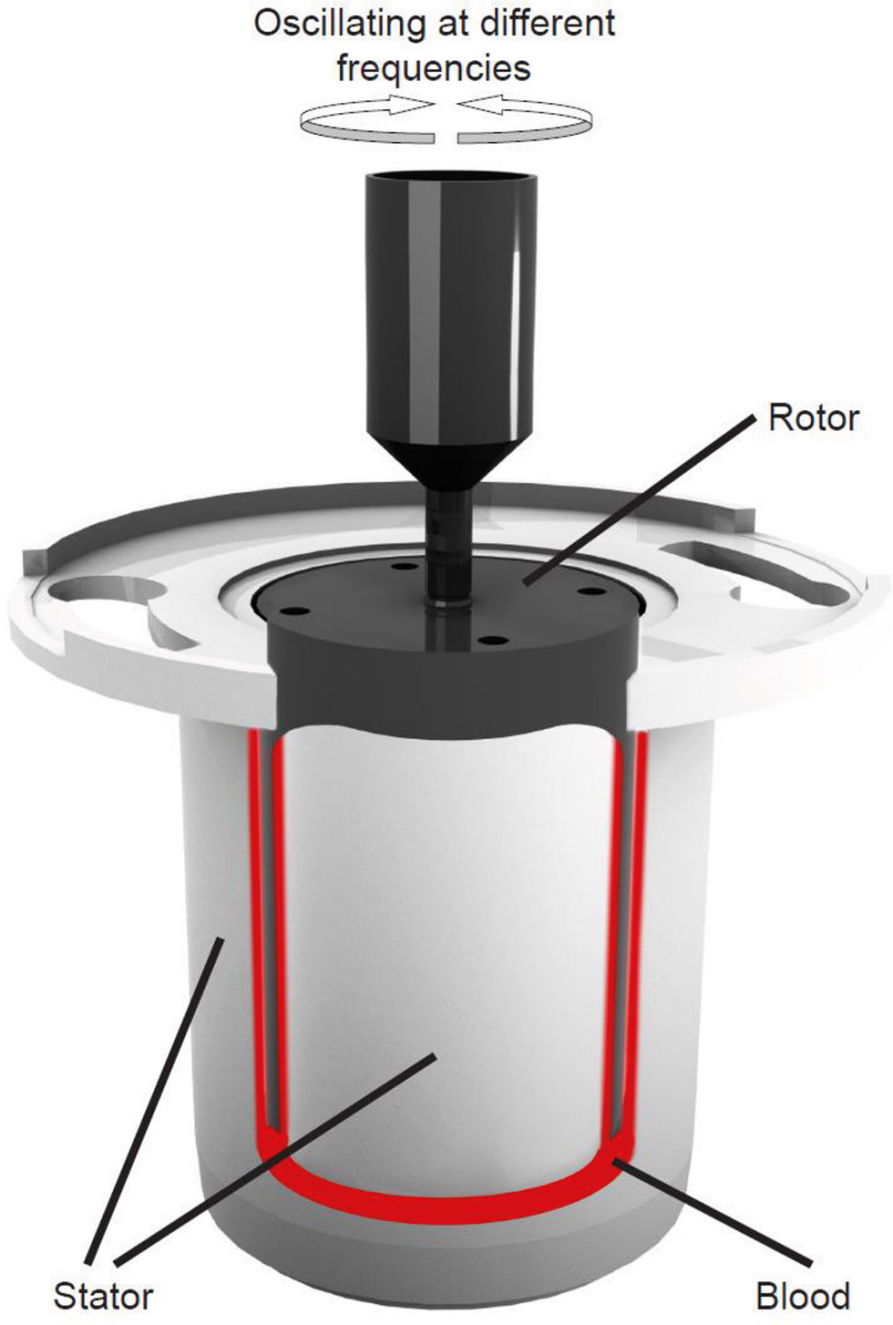
Diagram of a double-gap concentric cylinder measurement geometry. The double-gap geometry consists of a stationary cup or stator into which a 6.6 mL sample of blood is added after which a bob that is free to rotate called a rotor is then lowered into the sample. The movement of the rotor is controlled by an AR-G2-controlled stress rheometer and will oscillate at four different frequencies (0.20, 0.43, 0.93 and 2.00 Hz) sequentially over time.

**Figure 2:**
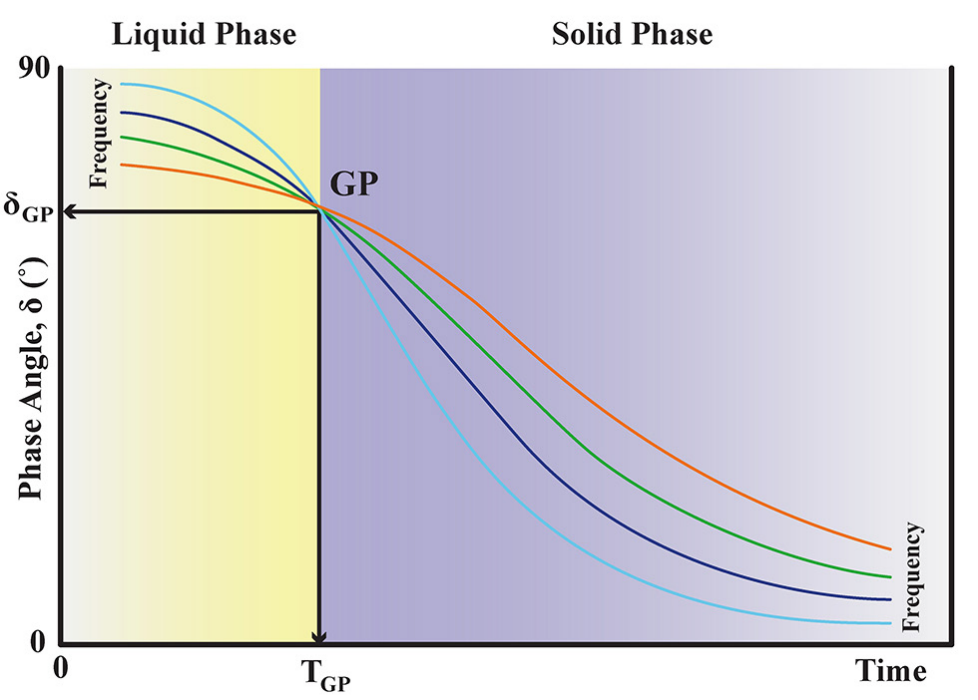
Gel point (GP) trace. This represents a typical GP result for one sample of blood. The illustration demonstrates how phase angle, δ, changes as coagulation progresses. δ has a range of 0° to 90°, where 90° identifies a purely viscous response and 0° identifies a purely elastic response with any value in between being a measure of the viscoelastic response to imposed stress. In a material that is changing from a liquid to a solid such as blood, there will be a decrease in δ. At the establishment of the incipient clot, when the clot becomes a viscoelastic solid, there is a point where the value of δ will be independent of frequency called the GP. The structural property of the incipient clot (in terms of its fractal dimension, d_f_) is derived from this frequency independent value of δ_GP_.

The viscoelastic measurements are based on attainment of the gel point (GP) from which the fractal dimension, d_f_, is determined.^9^ The GP technique has been previously validated for use with blood in several studies.^10–15^ Briefly, blood is placed within the double concentric measuring geometry of a controlled stress rheometer, AR-G2 (TA Instruments, New Castle, DE, USA) which is held a constant temperature of 37°C±0.1°C (figure 1). Immediately after loading the blood into the AR-G2, viscoelastic analysis is preformed using small amplitude oscillatory shear measurements at varying frequencies; 2, 0.93, 0.43 and 0.2 Hz, with an applied peak stress amplitude of 0.03 Pa. Repeatedly performing these measurements over time allows for the measurement of the GP (figure 2). The GP marks the transition of the blood from a viscoelastic liquid to a viscoelastic solid, where the GP identifies the formation of the incipient blood clot or the first point which a sample spanning (haemostatic) structure can be identified.^9^ In figure 2 the GP is located when the four frequencies cross-over. From the GP measurement we can quantify how the fibrin clot is organised by calculating its corresponding fractal dimension, d_f_.^9^

### Laboratory markers

A 4 mL aliquot of blood was drawn into tubes containing EDTA for Full Blood Count (FBC) analysis and then analysed using a Sysmex XE 2100 (Sysmex UK, Milton Keynes, UK). Parameters measured included: haemoglobin, haematocrit and platelet count. A 4.5 mL aliquot was collected into tubes containing citrate and then analysed using a Sysmex CA1500 (Sysmex UK, Milton Keynes, UK). Parameters measured included: prothrombin time, activated partial thromboplastin time, factor VIII and Clauss fibrinogen. D-dimer analysis was carried out using a latex immunoturbidimetric assay Hemosil HS D-dimer (Instrumentation Laboratory, Warrington, UK) with a ACL TOP 500 (Instrumentation Laboratory, Warrington, UK). Plasma cytokines (interleukin 6 and myeloperoxidase) were measured and quantified using a standard ELISA (Quantikine, R+D Systems, UK), according to the manufacturer’s instructions.

### Thrombin generation

Thrombin generation was measured using the Thrombin Generation Assay (TGA, Technoclone Diagnostics, Vienna, Austria). Plates were loaded into the fluorogenic plate reader TECAN infinite F200 pro (Labtech International, Uckfield, UK) and read every 60 s for 1 hour. TGA software was used to calculate individual thrombin generation curves.

### Platelet aggregation measurements

Measurement of platelet aggregation was achieved using the Multiplate analyser (Dynabyte GmBH, Munich, Germany). An aliquot of whole blood (3 mL) was transferred to hirudin tubes (Roche Diagnostics GmbH, Mannheim, Austria, Ref: 06675751) and kept at room temperature for 30 min before testing. Three hundred microlitres of whole hirudinated blood was added to 300 µL of saline preheated to 37°C and allowed to incubate for 3 min in individual test cells. Following incubation platelet activation was induced by addition of specific agonists to respective test cells, and electrical impedance was recorded. The agonists included ADP (20 µL of 0.2 mM stock solution) for measuring P2Y12 receptor aggregation, which is inhibited by clopidogrel and other thienopyridines. The second agonist was ASPItest reagent (20 µL of 15 mM stock solution) for measuring the inhibitory effect of aspirin.

### Statistical analysis

A power calculation was performed assuming a mean difference in d_f_ of 0.025 (based on pilot data) between unobstructive and obstructive CAD. Taking a SD of 0.045, a power of 0.85 and significance value set at 0.05 a minimum of 65 patients in both groups is required. With the study designed for consecutive patients and with a likely recruitment bias towards unobstructive CAD, we aimed to recruit double that number. Descriptive analyses were performed to establish baseline characteristics for both groups. Categorical variables are summarised using percentages and compared using χ^2^ tests while continuous variables are presented using mean and SD unless otherwise stated. Differences between groups were compared using two sample t-tests for parametric data or Kruskal-Wallis test for non-parametric data. Pearson correlation was undertaken to explore associations between d_f_ and demographic, laboratory markers and platelet aggregometry. Statistical analysis was performed using Minitab V.15 software (Havertown, Pennsylvania, USA) and deemed significant when p<0.05.

## Results

A total of 275 patients were recruited, full angiographic, viscoelastic measurements and platelet aggregation measurements were performed successfully in 251. Of the 251 patients recruited, 168 patients were classed as unobstructed CAD and 83 patients as obstructive CAD. The baseline characteristics and patient demographics for the two groups is recorded in table 1. Significant differences between the demographics of the two groups are observed for age (p=0.013), sex (p<0.001) and statin use (p=0.045). Results of the viscoelastic testing, laboratory markers and platelet aggregometry measurements can be found in table 2.

**Table 1:** Patient baseline characteristics and demographics

	Normal (n=80)	Unobstructed CAD (n=88)	Unobstructed (n=168)	Obstructive CAD (n=83)	p Value
Age	58.6±8.8	63.5±10.3	61.1±9.8	64.3±9.7	0.013
Sex (M:W)	32:48 (40%)	45:43 (51%)	77:91 (46%)	58:25 (70%)	<0.001
BMI	30.3±5.4	30.8±5.7	30.4±5.5	30.0±7.0	0.59
Smoking (current)	10 (12%)	14 (16%)	24 (14%)	14 (17%)	0.34
Hypercholesterol	54 (68%)	71 (81%)	125 (74%)	69 (83%)	0.31
HTN	40 (50%)	40 (45%)	90 (54%)	43 (52%)	0.82
DM	12 (15%)	29 (33%)	41 (24%)	16 (19%)	0.19
FHx MI	33 (42%)	47 (53%)	80 (48%)	39 (47%)	0.91
Antiplatelet use	60 (75%)	51 (58%)	111 (66%)	66 (78%)	0.11
Aspirin	58	34	92	57	
P2Y12	0	6	6	2	
Both	2	11	13	7	
Statins	50 (63%)	50 (63%)	109 (65%)	66 (80%)	0.045

All Caucasians but two. Other lowering lipid drugs: four ezetimibe and two fenofibrates.

BMI, body mass index; CAD, coronary artery disease; DM, diabetes mellitus; FHx MI, family history of stroke myocardial infarction; HTN, hypertension; M:W, men:women.

**Table 2:** Results of the viscoelastic testing, standard and specific markers for non-severe and severe CAD groups

	Normal (n=80)	Unobstructed CAD (n=88)	Unobstructed (n=168)	Obstructive CAD (n=83)	p Value
d_f_	1.728±0.052	1.735±0.053	1.732±0.052	1.749±0.057	0.028
IL-6	50.1±5.0	52.1±4.3	51.6±4.1	67.6±7.5	0.38
MPO	4541±1450	4563±1471	4544±1456	4636±1482	0.66
CRP	3.6±3.2	3.7±3.0	3.7±2.8	3.7±3.0	0.75
Hb (g/dL)	14.0±1.2	14.3±1.3	14.1±1.3	14.2±1.2	0.45
Plt (×10^9^/L)	263±67	253±69	258±67	249±53	0.22
HCT (g/g)	0.418±0.033	0.425±0.035	0.422±0.035	0.437±0.035	0.24
PT (s)	10.5±0.4	10.5±0.5	10.5±0.4	10.6±0.5	0.057
APTT (s)	25.8±2.1	25.8±1.9	25.8±2.0	26.2±1.9	0.13
FBG (g/L)	3.3±0.6	3.4±0.7	3.3±0.6	3.4±0.7	0.26
DD*	94 (IQR 71–147)	122 (IQR 81–178)	110 (IQR 74–167)	131 (IQR 80–209)	0.095
TG	137±44	119±50	128±76	136±93	0.51
FVIII	132±44	138±46	135±45	135±38	0.99
Multiplate ADP	85.3±32.2	77.1±27.5	81.4±29.8	76.9±28.5	0.24
Multiplate ASPI*	28 (IQR 13–70)	30 (IQR 18–83)	29.0 (IQR 15.8–78.1)	25.4 (IQR 18.0–64.5)	0.41

*Median and interquartile values reported.

APPT, activated partial thromboplastin time; CAD, coronary artery disease; CRP, C-reactive protein; DD, D-Dimer; FBG, fibrinogen; FVIII, Ffactor VIII; Hb, haemoglobin; HCT, haematocrit; IL-6, interleukin 6; MPO, myeloperoxidase; Plt, platelet count; PT, prothrombin time; TG, thrombin generation.

### Viscoelastic measurements

**Figure 3:**
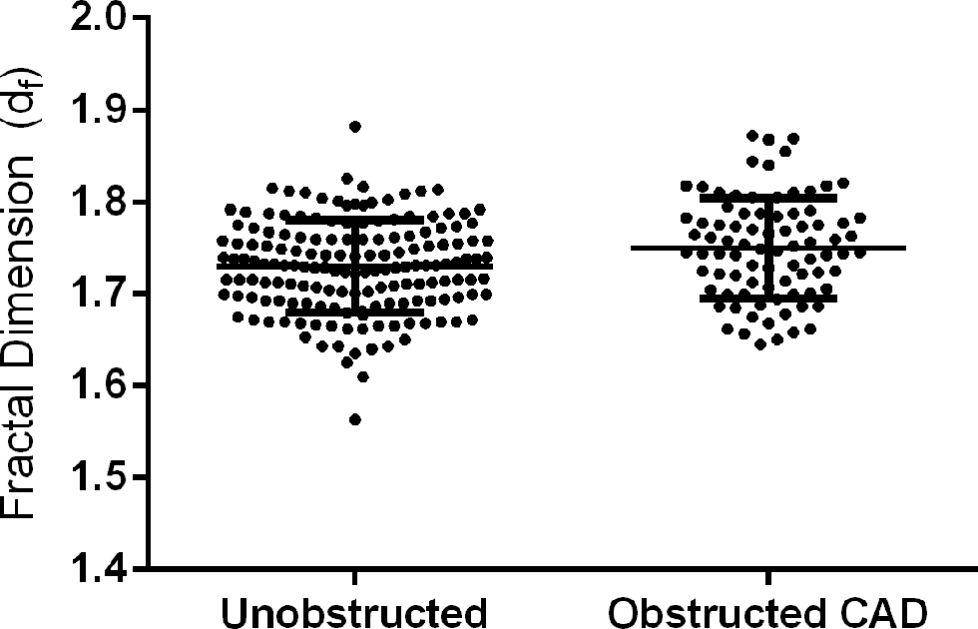
A graph showing the dispersion of d_f_ measurements among the unobstructed and the obstructed coronary artery disease (CAD) groups, where a significant increase in the value of d_f_ was observed for those patients with obstructive CAD when compared with unobstructed (d_f_=1.748±0.057 vs 1.732±0.052, p=0.028).

A significant increase in the value of d_f_ was observed for those patients with obstructive CAD when compared with unobstructed (d_f_=1.748±0.057 vs 1.732±0.052, p=0.028). We also preformed an analysis of covariance and analysis using a general linear model. This analysis demonstrated the difference in d_f_ between obstructive CAD and the unobstructed group remains significant (p<0.05) even when we adjust for fibrinogen concentration, haematocrit, antiplatelet function or the presence of hypercholesterolaemia. Furthermore, we analysed the data by dividing the unobstructed group into two separate groups, normal (n=80) and unobstructed CAD (n=88) giving total of three groups. Using a one-way analysis of variance (ANOVA; 95% CI) we observe a non-significant (p=0.053) increase in d_f_ when comparing the three groups: normal (n=80, d_f_=1.728±0.052); unobstructed CAD (1%<stenosis<50%) (n=88, d_f_=1.735±0.053) and obstructed CAD (≥50% stenosis) (n=83, d_f_=1.748±0.057). In addition, we reviewed the obstructed CAD group and classified it into 1, 2 and 3 vessel disease to assess whether there was a difference in d_f_. We found no significant difference in d_f_ depending on number of diseased vessels (p=0.83) (1 vessel (n=40) d_f_=1.738±0.060, 2 vessel (n=27) d_f_=1.770±0.055 and 3 vessel (n=14) d_f_=1.752±0.056). Dispersion of d_f_ for both the unobstructed and obstructed CAD groups can be seen in figure 3.

Comparing the d_f_ against patient demographics, we only found significant differences in men versus women, where d_f_ was raised in men (1.745±0.055 vs 1.723±0.052, p=0.007), and in smokers versus non/ex-smokers, where d_f_ was raised in smokers (1.761±0.037 vs 1.733±0.055, p=0.001). The differences observed in smokers versus non/ex-smokers persisted when studying the obstructive (1.754±0.050 vs 1.728±0.054, p=0.003) and unobstructive CAD (1.776±0.042 vs 1.745±0.059, p=0.043) groups.

### Laboratory markers and thrombin generation

We found no significant difference between unobstructed and obstructive CAD for any of the standard laboratory tests (see table 2). Positive correlations were observed between d_f_ and two of the laboratory markers: haematocrit (correlation coefficient=0.458, p=0.001) and fibrinogen concentration (correlation coefficient=0.242, p=0.001), where these relationships persisted when studying all patients recruited or in their individual obstructive (fibrinogen concentration: 0.308, p=0.004; haematocrit: 0.412, p=0.001) or unobstructive (fibrinogen concentration: 0.186, p=0.016; haematocrit: 0.477, p=0.001) CAD groups. We also reanalysed the laboratory markers data using one-way ANOVA (95% CI) to look at the data in three groups, namely normal, unobstructed CAD and obstructed CAD, however, we found no differences between the three groups.

### Platelet aggregometry

The use of antiplatelet therapy was only recorded when it was known to be effective in the patient. The effectiveness of antiplatelet therapy was determined from platelet aggregometry measurements taken at the same time as d_f_ measurements. Effective aspirin use is recorded by an ASPI reading of below 40IUs as deemed in a previous publication.^16^ Effective inhibition of P2Y12 receptor on the platelet is determined by an ADP value of below 47IUs.^17^ We found that 70% of patients were receiving some form of antiplatelet therapy with 89% of those receiving monotherapy and 11% dual antiplatelet therapy. Of the monotherapy group 95% were taking aspirin and 5% were taking a P2Y12 inhibitor. A significant positive correlation was observed between ASPI and d_f_ (0.157 p=0.014) but not ADP (0.089 p=0.34). We found a non-significant increase in d_f_ for those not taking antiplatelet therapy compared with those taking antiplatelet therapy (1.735±0.053 vs 1.746±0.053, p=0.12). A significant reduction in d_f_ was observed for patients on dual antiplatelet therapy compared with no therapy (1.722±0.053 vs 1.746±0.053, p=0.048).

## Discussion

**Figure 4:**
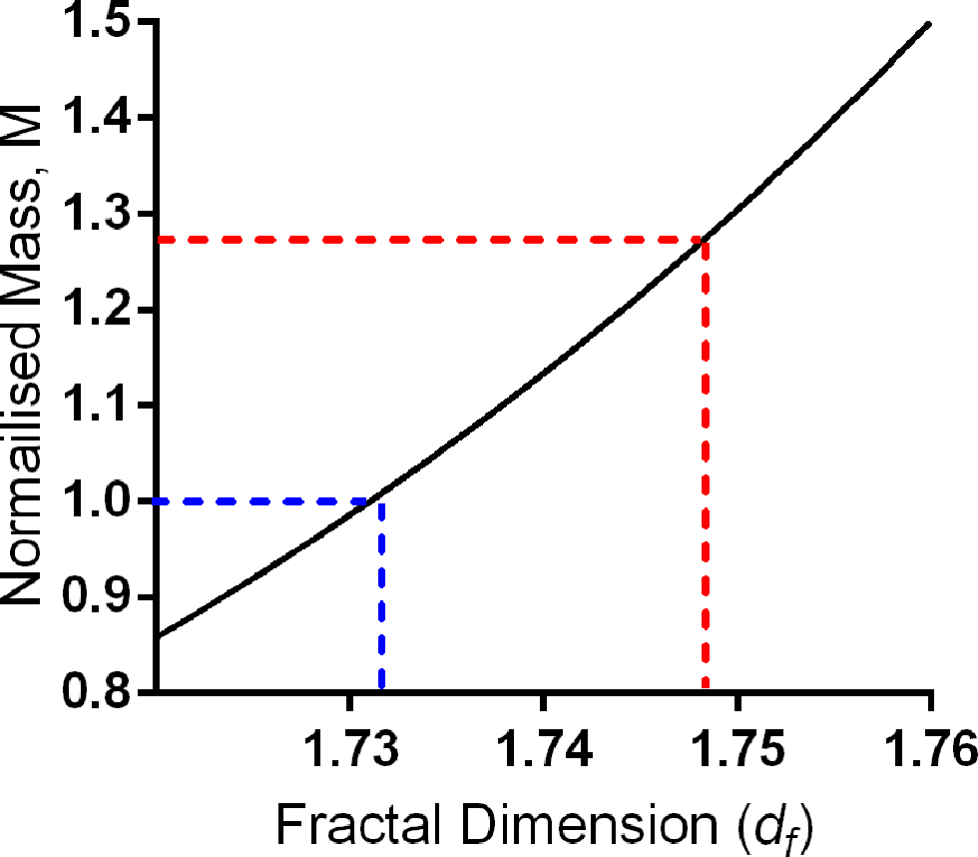
Fractal dimension (d_f_) versus mass. Graph showing the simplified non-linear relationship between d_f_ and the amount of mass incorporated into the incipient clot microstructure. Fibrin networks have fractal properties, where the mass, M, is related to d_f_ by the following power law equation (M≈ɛd_f_, where ɛ is some length scale value in the range 100 nm to 10 µm). The mass value on the y-axis is normalised to the unobstructed CAD value of d_f_=1.732, where at a d_f_ of 1.732 the normalised mass value is equal to 1 (blue dotted lines). The figure illustrates that a structure that forms with a d_f_ of 1.748, like the mean value of the obstructive CAD group (red dotted lines) would have an increased relative mass compared with a structure that formed with a d_f_ value of 1.732, having 1.27 times more mass.

This study for the first time shows that a whole blood biomarker that quantifies clot microstructure can be used to discriminate between those with unobstructed and those with obstructive CAD. We show that patients with obstructive CAD produce clots with a significantly higher d_f_ (1.748±0.057) when compared with those patients without obstructive CAD (1. 732±0.052) (p=0.028) (see figure 3). An increased value of d_f_ can be illustrated using a previously published computational model of a simplified branching network, which shows the relationship between d_f_ and the mass of the network.^18 19^ In figure 4 we show that a change in d_f_ from 1.732 (unobstructed CAD) to 1.748 (obstructive CAD) would correspond to an additional 25% more mass that would need to be incorporated into the structure of the incipient clot. Consequently, patients with obstructive CAD are more likely to produce clot microstructures that are consistently denser and more tightly packed, resulting in a clot that is of poorer quality, harder to breakdown and more likely to form a thrombus. This may be linked to the underlying hypercoagulable state of CAD patients suggested in previous studies.^1 2 20^

While a difference in the value of d_f_ is observed between the obstructive and unobstructive CAD groups, it is important to note that several patient demographics are also significantly different (see table 1). We found age was significantly different, where patients with unobstructed CAD were on average 3.2 years younger than the obstructive CAD group (61.1+9.8 years vs 64.3+9.7 years, respectively, p=0.013). This is perhaps unsurprising as age is a primary risk factor in advanced CAD.^21^ However, we found no significant correlations between age and d_f_ either in all patients or in their individual groups (all patients: correlation coefficient=−0.118, p=0.064; obstructive CAD: −0.210, p=0.055; unobstructive CAD: −0.105, p=0.164). Furthermore, in a previous study with a healthy control (70.6±7.1 years), there was no correlation between age and d_f_.^11^ The findings herein may be surprising considering studies have shown that increased age is often associated with an increase in certain haematological characteristics, such as fibrinogen concentration.^22^ Fibrinogen concentration has also been associated with cardiovascular risk; in the present study we did identify a positive correlation between d_f_ and fibrinogen concentration but fibrinogen was not significantly raised when comparing obstructive and unobstructive CAD.^23^ This suggests that while age is associated with advanced CAD, age itself is not the driving force behind the development of abnormal clot formation.

Sex was also significantly different between obstructive and unobstructive CAD, where there was a significantly higher proportion of men in the obstructive CAD group compared with unobstructed CAD group (70% vs 46%, p<0.001). This is unsurprising as CAD is often reported as more prevalent in men.^21^ When comparing d_f_versus sex, we found that d_f_ was significantly increased in men compared with women (1.746+0.055 vs 1.727+0.052, p=0.007). In the present study, the higher value of d_f_ in men may be due to the increased number of men with obstructive CAD compared with women, however, the raised d_f_ in men persists across both the obstructed (1.753±0.059 vs 1.737±0.053) and unobstructed (1.740±0.059 vs 1.725±0.053) CAD. Another rationale may be that men in general have an underlying propensity to form denser and more compact clots (hence higher d_f_s) than women. However, a previous study which used a healthy control (n=74) did not find a difference in the value of d_f_ between men and women.^11^ A previous study investigating myocardial infarction with d_f_ found that men were also more likely to have increased d_f_ values compared with women.^14^ Men with CAD have been shown to have a higher prevalence of experiencing an acute coronary syndrome or fatal coronary heart disease.^21^ A final explanation may be that men with CAD (either obstructive or unobstructive) are more likely to form denser more compact clots (hence higher d_f_s) than women, thus are at an increased risk of suffering an acute coronary syndrome.

Statin use was also found to be different when comparing the obstructive and unobstructive CAD groups. Statin use was significantly increased in the obstructive CAD group (80% vs 65%, p=0.045) (table 2). With the increased mean age and percentage of men in the obstructive CAD group, it is unsurprising that statin use is raised with both being strong indicators for statin use.^24 25^ A previous study has shown that statin use has a mediating effect on clot microstructure formation producing clots that are more open and porous compared with those formed in its absence.^26^ As a result, we would expect the group with the highest percentage of statin use to have the lowest value of d_f_, however, that is not the case. In the present study, the obstructive CAD group has around 80% (compared with 65% in the unobstructive CAD group) of patients taking statin while also having the highest value of d_f_. When comparing statin use and d_f_ for all patients recruited into the study, we found no significant difference between those taking and those not taking statins (1.736+0.055 vs 1.741+0.049, p=0.54). A possible reason being the wide range of different types of statin being used in the patients in this study, which may have differing effects alongside the varying comorbidities and heterogeneous nature of the patient population.

A significant increase in d_f_ was also observed in smokers compared with non-smokers/ex-smokers (1.761±0.037 vs 1.733±0.055, p=0.001). While the number of smokers and non/ex-smokers was not significantly different between the obstructive and unobstructive CAD groups, smoking has been suggested to contribute to the formation of hypercoagulable conditions which could account for its relationship with d_f_.^27^

In this study, we found that antiplatelet use was not significantly different between the obstructive and unobstructive CAD groups (78% vs 66%, p=0.11). We found that when comparing effective antiplatelet (either mono or dual) therapy against non-effective/no antiplatelet therapy, with respect to d_f_, patients with effective antiplatelet therapy had a non-significant decrease in the value of d_f_ (1.735±0.053 vs 1.746±0.053, p=0.11). We have previously shown that mono-antiplatelet therapy with aspirin can lower d_f_ in patients with acute vascular disease, however, this was in a dose of 300 mg of aspirin, not 75 mg, and the effect was only an acute lowering of d_f_ which returned to baseline levels after 24 hours.^11^ Despite the fact that monotherapy did not significantly reduce the value of d_f_ in the present study, we did find a significant positive correlation (0.157, p=0.014) between d_f_ and the ASPI test (Multiplate) results showing that the stronger the inhibition of the aspirin the lower the value of d_f_. Of those patients receiving antiplatelet therapy, 12% were receiving dual antiplatelet. We found a significantly reduced value of d_f_ in those receiving dual antiplatelet therapy compared with no therapy (1.722±0.053 vs 1.746±0.053, p=0.048). These results suggest that dual antiplatelet therapy is effective in mediating clot microstructure, where patients have lower values of d_f_ corresponding to the production of less densely packed and more porous clots.^4^ This study provides further evidence of the potential of d_f_ in monitoring the effects of antiplatelet therapy on clot microstructure formation.^11^

The limitations of this study include discrepancy in the numbers collected for both groups. As the study was designed to collect consecutive patients admitted for a routine diagnostic angiography, it was not possible to collect matched patients for both groups. This has resulted in twice as many patients with non-obstructive CAD being recruited (n=168 vs n=83). While this reduces the power of any statistical analysis, the numbers recruited are still sufficient for meaningful interpretation of the results. Finally, the presence of a 50% stenosis on invasive coronary angiography was used to define the presence of obstructive CAD. This is an accepted gold standard with prognostic implications. However, intravascular imaging was not used routinely so it is possible that some of the patients with angiographic irregularities or lesions <50% may have contained positively remodelled plaques with potentially vulnerable morphology. It is not clear how the presence of these plaques might effect d_f_ and requires further study.

In this study, we have performed the first characterisation of clot microstructure in patients with stable CAD. We have shown that in patients investigated for stable chest pain, the presence of obstructive CAD is associated with an increased potential to produce denser more compact clots and unfavourable morphology. We have also identified that men with obstructive CAD disease produce unfavourable clot microstructures compared with women. Additionally, patients with CAD receiving dual antiplatelet therapy have a reduced d_f_ value linked to a more favourable looser less compact clot. The d_f_ measurement can potentially be used to risk-stratify patients with stable CAD and has the potential to assess the efficacy of therapeutic interventions by measuring changes in clot microstructure.
